# Changes in the interstitial cells of Cajal and neuronal nitric oxide synthase positive neuronal cells with aging in the esophagus of F344 rats

**DOI:** 10.1371/journal.pone.0186322

**Published:** 2017-11-28

**Authors:** Hee Jin Kim, Nayoung Kim, Yong Sung Kim, Ryoung Hee Nam, Sun Min Lee, Ji Hyun Park, Daeun Choi, Young-Jae Hwang, Jongchan Lee, Hye Seung Lee, Min-Seob Kim, Moon Young Lee, Dong Ho Lee

**Affiliations:** 1 Department of Internal Medicine, Seoul National University Bundang Hospital, Seongnam, S. Korea; 2 Department of Internal Medicine, Myongji Hospital, Goyang, S. Korea; 3 Department of Internal Medicine and Liver Research Institute, Seoul National University College of Medicine, Seoul, S. Korea; 4 Department of Gastroenterology and Digestive Disease Research Institute, Wonkwang University School of Medicine, Iksan, S. Korea; 5 Department of Pathology, Seoul National University Bundang Hospital, Seongnam, S. Korea; 6 Department of Physiology and Institute of Wonkwang Medical Science, Wonkwang University College of Medicine, Iksan, S. Korea; University of Nevada School of Medicine, UNITED STATES

## Abstract

The aging-associated cellular and molecular changes in esophagus have not been established, yet. Thus we evaluated histological structure, interstitial cells of Cajal (ICCs), neuronal nitric oxide synthase (nNOS)-positive cells, and contractility in the esophagus of Fischer 344 rat at different ages (6-, 31-, 74-weeks, and 2-years). The lamina propria thickness and endomysial area were calculated. The immunoreactivity of c-Kit, nNOS and protein gene product (PGP) 9.5 was counted after immunohistochemistry. Expression of *c-Kit*, *stem cell factor* (SCF), *nNOS* and *PGP 9*.*5* mRNA was measured by real-time PCR, and expression of c-Kit and nNOS protein was detected by Western blot. Isovolumetric contractile force measurement and electrical field stimulation (EFS) were conducted. The lamina propria thickness increased (6 week *vs* 2 year, *P* = 0.005) and the endomysial area of longitudinal muscle decreased with aging (6 week *vs* 2 year, *P*<0.001), while endomysial area of circular muscle did not significantly decrease. The proportions of NOS-immunoreactive cells and c-Kit-immunoreactive areas declined with aging (6 week *vs* 2 year; *P*<0.001 and *P* = 0.004, respectively), but there was no significant change of PGP 9.5-immunopositiviy. The expressions of *nNOS*, *c-Kit* and *SCF* mRNA also reduced with aging (6 week *vs* 2 year; *P* = 0.006, *P* = 0.001 and *P* = 0.006, respectively), while the change of PGP 9.5 mRNA expression was not significant. Western blot showed the significant decreases of nNOS and c-Kit protein expression with aging (6 week *vs* 2 year; *P* = 0.008 and *P* = 0.012, respectively). The EFS-induced esophageal contractions significantly decreased in 2-yr-old rat compared with 6-wk-old rats, however, L-NG-Nitroarginine methylester did not significantly increase the spontaneous and EFS-induced contractions in the 6-wk- and 2-yr-old rat esophagus. In conclusion, an increase of lamina propria thickness, a decrease of endomysial area, c-Kit, SCF and NOS expression with preserved total enteric neurons, and contractility in aged rat esophagus may explain the aging-associated esophageal dysmotility.

## Introduction

Aging affects the structure and function of gut as a result of accumulation of diverse deleterious cellular and biochemical changes [1]. Several human studies reported aging-associated changes in the esophageal motility such as increased esophageal stiffness, reduced peristaltic function [2] and decreased lower esophageal sphincter (LES) relaxation [3, 4]. A recent human research showed decreased upper esophageal sphincter pressure, distal esophageal motility and peristaltic velocity with advancing age using high resolution esophageal manometry [5]. These changes may be a major contributor to dysphagia and reflux symptom which are commonly reported in elderly individuals [6, 7].

The cellular and molecular changes exhibited by the aging gut cells vary. Aged intestinal smooth muscle cells (SMCs) exhibit a number of changes in the signaling pathways that regulate contractions [8, 9]. Researches on aging enteric neurons reported a neuronal loss or degeneration in the aged gut [8, 10–13]. The extent of enteric neuronal loss during aging is likely to be variable in different studies. Specifically, different neuronal subpopulations may be differentially affected. Several studies have provided evidence that excitatory cholinergic neurons were reduced in number in older animals, whereas inhibitory nitrergic neurons have been reported to be spared [14–18]. However, our group reported a decrease of nitrergic neurons in the colon [19] and stomach [20] of aged F344 rats. In addition, there have been evidences for age-related enteric neurodegeneration. Swollen and dystrophic nerve fibers have been described in the aging rat [15] and mouse [17].

Interstitial cell of Cajal (ICC) plays a pivotal role as the pacemakers in the control of gastrointestinal (GI) motility. They generate electrical pacemaker activity that provides the musculature with the mechanism to produce propulsive rhythmic contractile activity [21, 22] and they appear to be conduits for nitrergic innervation of muscle [23]. ICCs express *c-Kit*, which is a membrane receptor with tyrosine kinase activity [24], and stem cell factor (SCF) serves as a ligand of Kit. It is well established that ICCs survival and function depend on the activation of c-Kit [25]. In spite of the role that ICCs paly in the control of motility, very few studies have investigated changes in properties or numbers of ICCs in aging. Gomez-Pinilla *et al*. showed the number and volume of ICC networks in the normal human stomach and colon decline with aging using immunohistochemistry (IHC) analysis [26] and recently our group also reported a decrease of ICC expressions with aging in the stomach of F344 Fischer rats using IHC, real-time PCR and Western immunoblotting [20].

The majority of research on aging of GI tract has been carried out in the stomach, small intestines and colons, and to the best of our knowledge, there have been only two early studies on the effect of aging on esophagus which reported total neuronal loss in rat and human, respectively [27, 28]. No data address the effect of age on ICCs of esophagus. From this background, the aim of this study is to assess the changes of histological structure and expressions of ICCs, neuronal nitric oxide synthase (nNOS)-immunoreactive neurons with SCF in the esophagus of 6-, 31-, 74-wk and 2-yr aged F344 rats, which are equivalent to 5, 30, 60 and 80 years of human age, respectively. In addition, esophageal contractility was compared between young and old aged rats using electrical field stimulation (EFS).

## Materials and methods

### Animals and tissue preparation

Specific pathogen-free, male Fischer 344 rats (Orient Co. Ltd., Seoul, Korea), 6-, 31-, and 74- week and 2- year of age were used for the experiments (each, n = 6). The animals were housed in a cage maintained at 23°C, with 12:12-h light-dark cycles under specific pathogen-free conditions. The rats were fasted but allowed water for 12 h before the experiments. All rats were euthanized by carbon dioxide after experiments approved in our IACUC Animal Care and Use Protocol. 1 cm length of the distal esophagus per rat was obtained, and fixed in 10% buffered formalin for histology. The specimens were embedded in paraffin, sectioned perpendicularly to the lumen (section thickness, 4 μm) and stained with hematoxylin and eosin (H&E). All experimental procedures described here were approved by the Institutional Animal Care and Use Committee of Seoul National University Bundang Hospital (BA1403-148/012-02).

### Histology

The histology was analyzed following the methods of our previous reports [19, 20]. The histology was evaluated by a pathologist (H. S. Lee) blinded to the age of the animal. One H&E-stained slide per rat (n = 6 for each age group) and four to five fields per slide were randomly selected and checked under 40×objective lens. The thicknesses of the total esophageal wall and lamina propria were quantified using the Image- Pro Plus analysis system (Media Cybernetics, SanDiego, CA). The thickness of lamina propria was expressed as a percentage of thickness of the total esophageal wall. The areas of the circular muscle (CM) endomysium and longitudinal muscle (LM) endomysium were quantified using the Image- Pro Plus analysis system (Media Cybernetics, SanDiego, CA). The endomysial area was expressed as a percentage of total muscle area.

### Immunohistochemistry for c-Kit, nNOS, protein gene product (PGP) 9.5

The immunohistochemistry was performed following the methods of our previous reports [19, 20]. The tissue sections were incubated with the following primary antibody: anti-nNOS antibody (dilution 1:500; AB5380 Chemicon Millipore Corporation, Billerica, MA, USA), anti-c-Kit antibody (dilution 1:100; polyclonal rabbit anti-human CD117, DAKO, Glostrup, Denmark) and anti-PGP 9.5 antibody (dilution 1:250; CM 329 AK; Biocare Medical, Concord, CA) after deactivation of endogenous peroxidase with 3% hydrogen peroxide and blocking of nonspecific binding sites. The immunostaining was performed using an automatic immunostainer (BenchMark XT, Ventan Medical Systems, Inc., Tucson, AZ, USA) according to the manufacturer’s instructions. An UltraView Universial DAB detection Kit (Ventana Medical Systems) was used as secondary antibody. The negative control for immunohistochemistry was performed without primary antibody. The immunostained tissues were examined under a light microscope (Carl Zeiss, Jena, Germany) linked to a computer-assisted image analysis system (AxioVision Rel.4.8; Carl Zeiss). Two immunostained slides per each rat (n = 6 for each age group) were prepared, and four to five fields per slide were randomly selected to obtain micrographs at x1000 for nNOS, PGP 9.5, c-Kit and PGP 9.5. Mast cells, known to express c-Kit were excluded by their round or oval shape and lack of processes were excluded from counts [26, 29, 30]. Quantitative assessment of c-Kit, nNOS and PGP 9.5 immunoreactivity was performed using the Image- Pro® Plus analysis system (Media Cybernetics, SanDiego, CA, USA). c-Kit- positive areas expressed as a percentage of c-Kit-positive areas/total areas. nNOS-positive cells expressed as a percentage of number of nNOS-positive cells/total cells. Likewise, PGP 9.5-positive cells and areas expressed as a percentage of number of PGP 9.5-positive cells/total cells and PGP 9.5-positive areas/total areas.

### Real-time PCR for c-Kit, SCF and nNOS

*c-Kit*, *SCF* and *nNOS* mRNA levels were measured by real-time PCR (q-PCR) according to the method in our previous reports [19, 20]. RNA was extracted from the esophageal muscle tissues devoid of the mucosa, submucosa and preferably serosa using an RNeasy Plus Mini Kit (Qiagen, Valencia, CA, USA) according to the manufacturer’s instructions. RNA samples were diluted to a final concentration of 0.5 mg/mL in RNase-free water and stored at -80°C until use. Synthesis of the cDNA was performed with 1 mg of total RNA with M-MLV reverse transcription reagents (Invitrogen, Carlsbad, CA, USA). The 20 μL reverse transcription reaction consisted of 4 μL of first-strand buffer, 500 mM deoxynucleoside triphosphate mixture, 2.5 mM oligo (dT) 12–18 primer, 0.4 U/mL ribonuclease inhibitor, and 1.25 U/mL Moloney murine leukemia virus 152 reverse transcriptase (Invitrogen). The thermal cycling parameters for the reverse transcription were 10 minutes at 65°C, 50 minutes at 37°C and 15 minutes at 70°C. Real-time PCR amplification and determination were performed using SYBR Premix Ex TaqTM (Takara Bio, Shiga, Japan) according to the manufacturer's protocols. The following primers were used: *c-Kit* forward, TTC CTG TGA CAG CTC AAA CG; *c-Kit* reverse, AGC AAA TCT TCC AGG TCC AG; *SCF* forward, CAA AAC TGG CGA ATC TT; *SCF* reverse, GCC ACG AGG TCA TCC ACT AT; *nNOS* forward, CTA CAA GGT CCG ATT CAA CAG; *nNOS* reverse, CCC ACA CAG AAG ACA TCA CAG; *GAPDH* forward, AGG TGA AGG TCG GAG TCA; and *GAPDH* reverse, GGT CAT TGA TGG CAA. The *GAPDH* gene was used as an endogenous reference as a control for expression that was independent of sample-to-sample variability. The amplification protocol consisted of an initial denaturation step at 95°C for 10 seconds, followed by 40 cycles of denaturation for 5 seconds at 95°C and annealing/extension of 33 seconds at 55°C. The relative expression levels of target genes were normalized by dividing the target Ct values by the endogenous Ct values. All equipment was purchased from Applied Biosystems and used according to their protocols. RNA-free water was used in real-time PCR for the no-template control (NTC). After amplification, we performed melting curve analysis using ABI PRISM® 7000 Sequence Detection System software (Applied Biosystems).

### Western blotting for c-Kit and nNOS

c-Kit and nNOS protein levels were measured by western blotting according to the method in our previous report [20]. The esophageal muscle tissue devoid of mucosa, submucosa and preferably serosa was homogenized with lysis buffer containing 25 mM Tris-HCL (pH 7.4), EGTA (1 mM), DTT (1 mM), leupeptin (10 μg/mL), aprotinin (10 μg/mL), PMSF (1 mM), and Triton X-100 (0.1%). Briefly, the proteins (each sample, 100 μg) were separated by SDS–PAGE (8% wt/wt gel) and transferred to PVDF membranes. All procedures were performed in Tris buffer (40 mM, pH 7.55) containing 0.3 M NaCl and 0.3% Tween 20. The membranes were then blocked with dried milk (5% wt/vol) and subsequently incubated with c-Kit (1:100; rabbit polyclonal antibody, Santa Cruz Biotechnology, Santa Cruz, CA, USA), nNOS (1:500; mouse monoclonal IgG2a antibody, BD Biosciences, San Diego, CA, USA) and β-actin (1:1000; rabbit polyclonal antibody, Biovision, Milpitas, CA, USA) at 4°C overnight. The blots were incubated with secondary antibody (rabbit polyclonal antibody, Santa Cruz Biotechnology for c-Kit (dilution 1:500) and β-actin (dilution 1:1000) and mouse polyclonal antibody (1:1000; Santa Cruz Biotechnology) for nNOS, and an imaging analyzer was used to measure the band densities. For c-Kit immunoblot, each of the optical density of the mature (145 kDa) and immature (120 kDa) forms was combined into one in the analysis process using densirometry [31].

### Isometric force measurements and EFS

Mechanical responses were performed to investigate the functional difference of esophagus between 6-wk- and 2-yr-old F344 rats using standard organ-bath techniques. Segments of the distal esophagus were removed through a midline chest incision and opened. Luminal contents were removed by a washing with Krebs-Ringer bicarbonate solution (KRB), and the mucosa was removed leaving the tunica muscularis and remnants of the submucosa. The muscles strips, oriented in the axis of the circular muscle, were mounted under 1 g tension and then allowed to equilibrate for 1–2 h with constant perfusion with fresh KRB, spontaneous contractile activity was measured by area under the curve (AUC) at the resting state. For inducing muscle contractile response, electrical field stimulation (EFS) was applied through a pair of platinum electrodes placed on either side of esophageal muscle strips with frequency of 10, 20 and 40 Hz for 60 s at intervals of 10 minutes. The contractile responses by EFS of esophageal muscle were compared in the absence or presence of L-NG-Nitroarginine methyl ester (L-NAME, 10 μM). We assessed the differences of contractile activity by quantifying excitation response during EFS. All data were recorded using the PowerLab data acquisition system. Using the recorded waveform, the AUC was calculated by integrating the differences between the maximum and minimum values obtained immediately before and after the EFS stimulation. When the percent changes of EFS response under drug L-NAME treatment were measured, the value before the drug treatment was defined as 100%.

### Statistical analysis

All statistical calculations were performed using SPSS software (version 20.0; SPSS Inc., Chicago, IL, USA). The results were compared by the Mann-Whitney U-test or Kruskal–Wallis test. All values are reported as means ± standard error. Statistical significance was set at a P value < 0.05.

## Results

### Influence aging on lamina propria thickness and endomysial area

**Fig 1:**
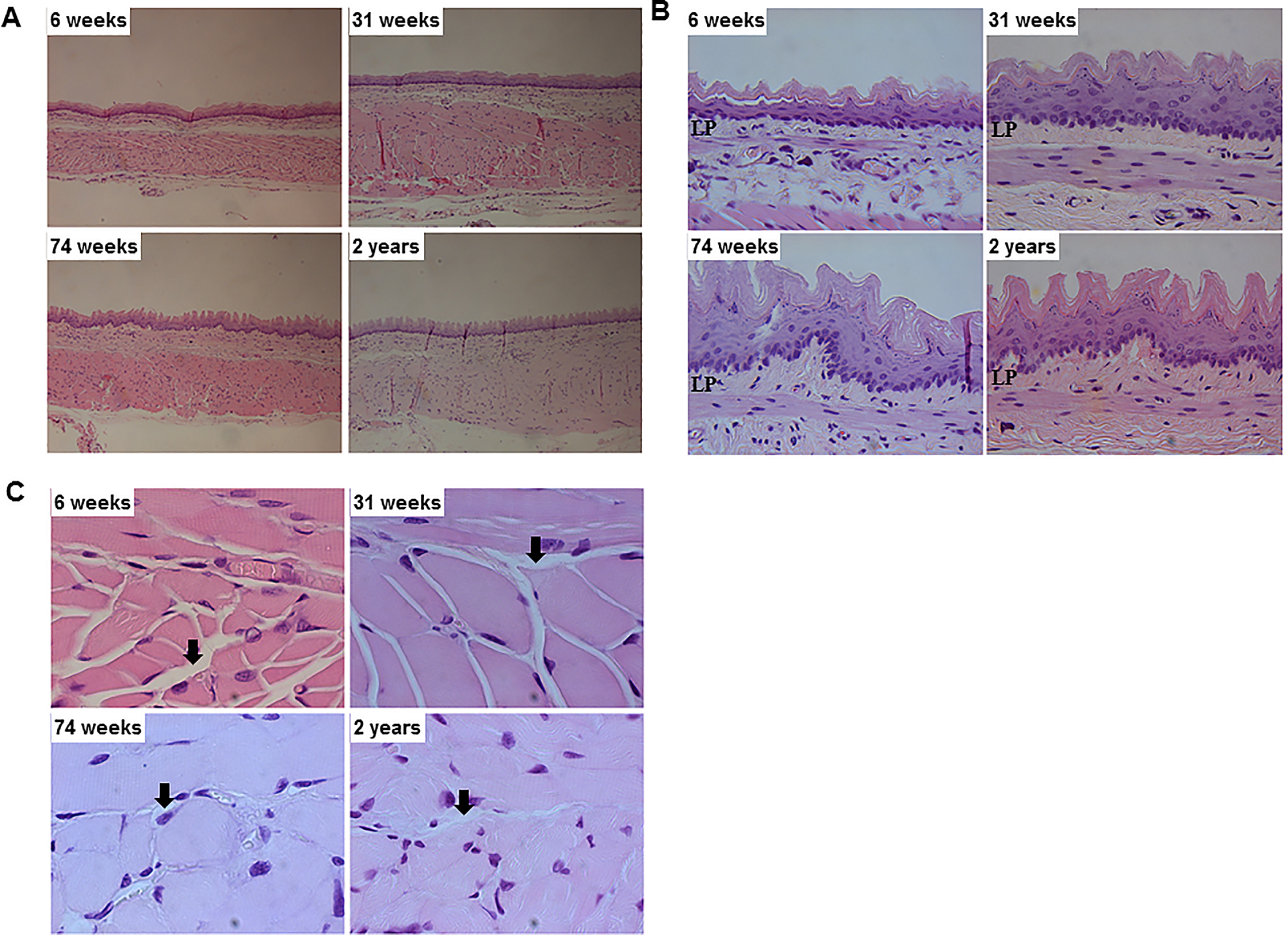
The change of esophageal structure with aging. (A) Total layer of esophagus demonstrated by hematoxylin and eosin (H&E) staining (x200 magnification). (B) Lamina propria layer demonstrated by H&E staining (x400 magnification) (C) Endomysial area demonstrated by H&E staining (x1000 magnification) in 6-, 31-, 74-wk-, and 2-yr-old rats (each group, n = 6). Arrows indicate the endomysium. LP means lamina propria.

**Fig 2:**
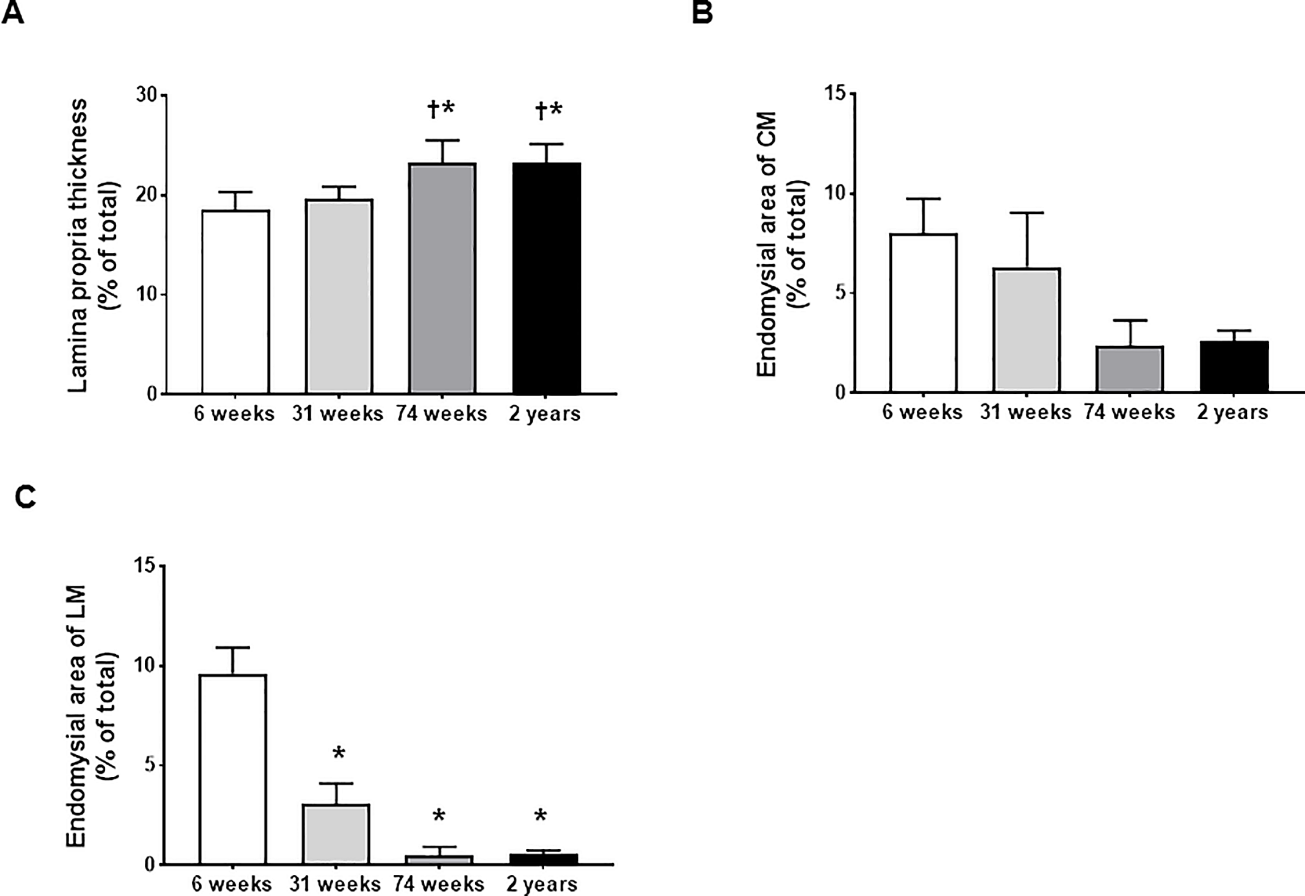
The changes in thickness of lamina propria and endomysial area of rat esophagus with aging. Thickness of lamina propria (A), endomysial area of circular muscle (B) and endomysial area of longitudinal muscle (C) in 6-, 31-,74-wk-, and 2-yr-old rats (each group, n = 6). The results are presented as means ± standard error. *P < 0.05 compared with 6 week of age; ^†^*P* < 0.05 compared with 31 week of age.

Representative images of total layer, lamina propria layer and endomysial area of rat esophagus stained by H&E staining at four different ages are shown in Fig 1. In detail, the average thickness of lamina propria in the 74-wk and 2-yr-old rats significantly increased compared with 6- and 31-wk-old rats (6-wk *vs* 74-wk, *P* = 0.005; 6-wk *vs* 2-yr, *P* = 0.005; 31-wk *vs* 74-wk, *P* = 0.029; 31-wk *vs* 2-yr, *P* = 0.03) (Table 1 and Fig 2A). The endomysial area of CM showed the tendency of decrease with aging, but not statistically significant (6-wk *vs* 74-wk, *P* = 0.137; 6-wk *vs* 2-yr, *P* = 0.135) (Table 1 and Fig 2B). However, the endomysial area of LM significantly decreased with aging. The average endomysial area of LM in the 31- wk, 74-wk and 2-yr-old rats was significantly smaller than that in the 6-wk-old rats (6-wk *vs* 31-wk, *P* <0.001; 6-wk *vs* 74-wk 0.50 ± 0.43, *P* <0.001; 6-wk *vs* 2-yr, 0.58 ± 0.16, *P* <0.001) (Table 1 and Fig 2C).

**Table 1:** The proportions of lamina propria thickness and endomyisal area of esophagus in 6-, 31-, 74-wk- and 2-yr-old rats.

	6 weeks	31 weeks	74 weeks	2 years	*P*-value		
					6 wk *vs* 74 wk	6 wk *vs* 2 yr	31 wk *vs* 2 yr
Lamina propria thickness per total thickness (%)	18.55 ± 1.76	19.69 ± 1.14	23.22 ± 2.30	23.20 ± 1.91	**0.005**	**0.005**	**0.03**
Endomysial areas per total circular muscle areas (%)	8.00 ± 1.73	6.32 ± 2.72	2.37 ± 1.27	2.61 ± 0.52	0.137	0.135	0.427
Endomysial areas per total longitudinal muscle areas (%)	9.62 ± 1.29	3.08 ± 1.03	0.50 ± 0.43	0.58 ± 0.16	**<0.001**	**<0.001**	0.221

The data are expressed as mean ± SD.

Bold style means the statistical significance.le means the statistical significance.

### Influence aging on nNOS- and PGP 9.5-immunoreactive neuron

**Fig 3:**
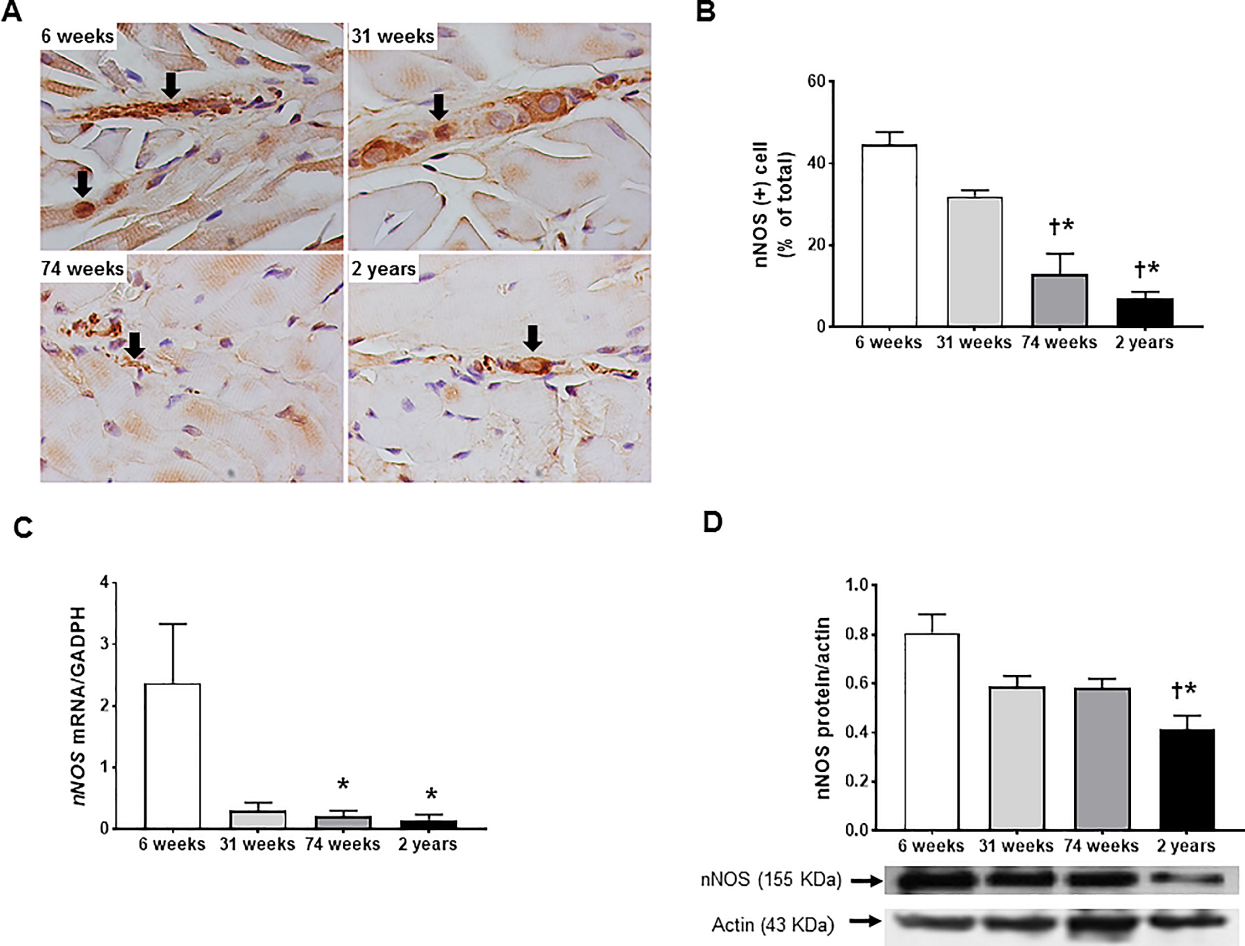
The changes in neuronal nitric oxide synthase (nNOS) immunoreactivity and the expression of *nNOS* mRNA and nNOS protein of rat esophagus with aging. Representative images of nNOS-immunoreactive cells in muscular layer (A), nNOS immunohistochemistry (B) in 6-, 31-, 74-wk-, and 2-yr-old rats (each group, n = 6). nNOS immunohistochemistry was expressed as percentage of the number of nNOS-immunoreactive cells per total cells. Arrows indicate the nNOS-immunoreactive cells (x1000 magnification). Expression of *nNOS* mRNA measured by real time-PCR (C) and nNOS protein measured by western blot (D) in 6-, 31–74-wk-old rats (each, n = 7) and 2-yr-old rats (n = 10). Each bar represents the mean ± standard error. *P < 0.05 compared with 6 week of age; ^†^*P* < 0.05 compared with 31 week of age.

**Fig 4:**
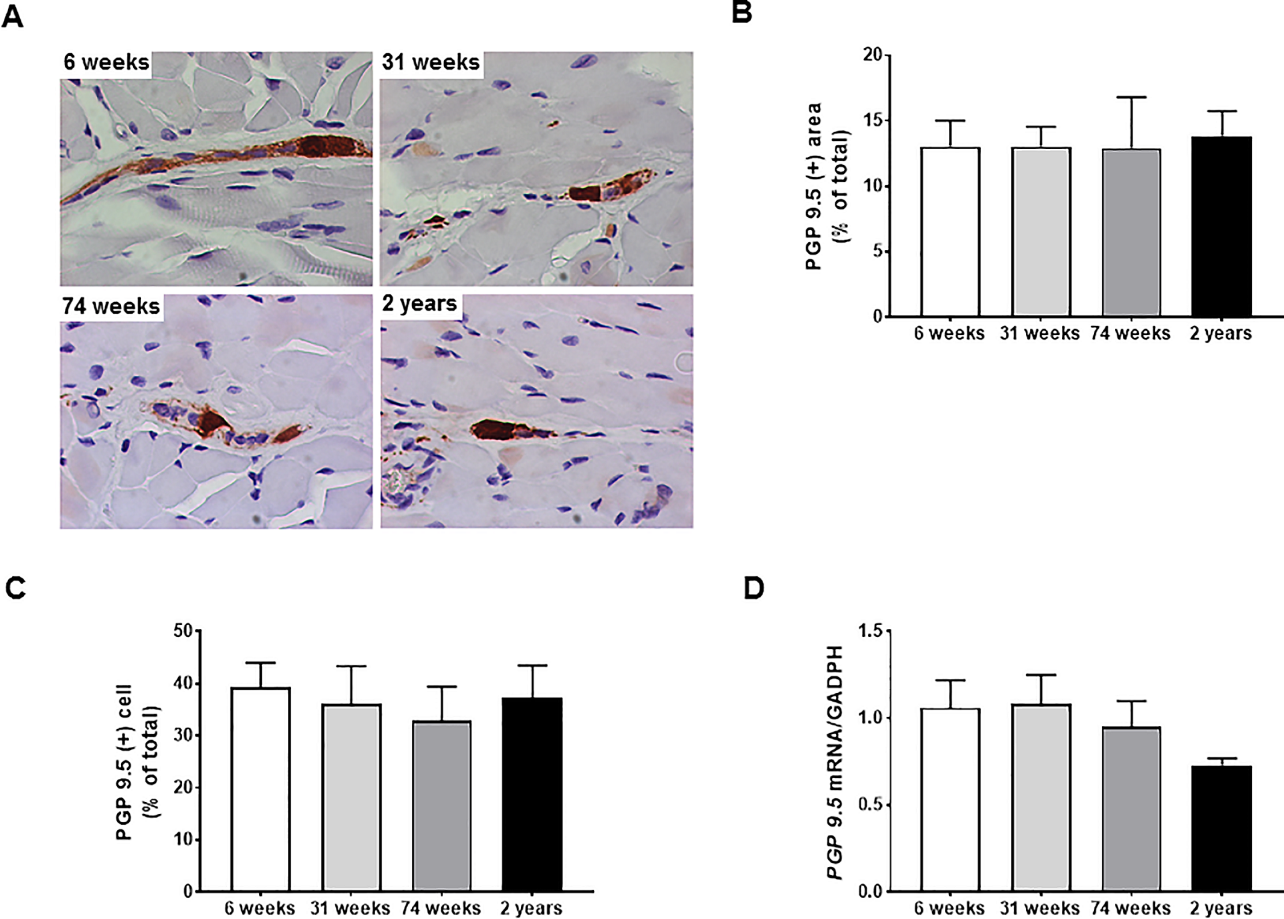
The changes in protein gene product (PGP) 9.5 immunoreactivity and the expression of *PGP 9*.*5* mRNA of rat esophagus with aging. Representative images of PGP 9.5-immunoreactive cells in myenteric plexus (x1000 magnification) (A), PGP 9.5 immunohistochemistry expressed as percentage of PGP 9.5-immunoreactive cells per total cells (B), PGP 9.5 immunohistochemistry expressed as percentage of PGP 9.5-immunoreactive areas per total areas (C), the expression of *PGP 9*.*5* mRNA measured by real time-PCR (D) in 6-, 31–74-wk-old rats (each, n = 7) and 2-yr-old rats (n = 10). Each bar represents the mean ± standard error.

Fig 3A shows representative images of nNOS-immunoreactivity in muscular layer of rat esophagus with four different ages. The proportion of nNOS-immunoreactive cells to total cells in the 74-wk- and 2-yr-old rats decreased compared with that of the 6- and 31-wk-old rats (6-wk *vs* 74-wk, *P* <0.001; 6-wk *vs* 2-yr, *P* <0.001; 31-wk *vs* 74-wk, *P* = 0.001, 31-wk *vs* 2-yr, *P* <0.001) (Table 2 and Fig 3B). Similar findings are shown in the expression of mRNA and protein. *nNOS* mRNA expression in the 74-wk and 2-yr-old rats declined compared with that of the 6-wk-old rats (6-wk *vs* 74-wk, 2.38 ± 0.95 *vs* 0.2 ± 0.1, *P* = 0.048; 6-wk *vs* 2-yr, 2.38 ± 0.95 *vs* 0.15 ± 0.09, *P* = 0.006) (Fig 3C), and nNOS protein expression in the 2-yr-old rats decreased compared with that of the 6- and 31-wk-old rats (6-wk *vs* 2-yr, 0.806 ± 0.078 *vs* 0.413 ± 0.057, *P* = 0.008; 31-wk *vs* 2-yr, 0.589 ± 0.042 *vs* 0.413 ± 0.057, *P* = 0.039) (Fig 3D). In contrast, changes in PGP 9.5-immunoreactivty and *PGP 9*.*5* mRNA with aging were not significantly different (Table 2 and Fig 4).

**Table 2:** The immunoreactivities of nNOS, PGP 9.5 and c-Kit of esophagus in 6-, 31-, 74-wk- and 2-yr-old rats.

	6 weeks	31 weeks	74 weeks	2 years	*P* value		
					6 wk *vs* 74 wk	6 wk *vs* 2 yr	31 wk *vs* 2 yr
nNOS-positive cells per total cells (%)	44.65 ± 3.10	32.03 ± 1.47	12.95 ± 5.03	7.09 ± 1.61	**<0.001**	**<0.001**	**<0.001**
PGP 9.5-positive areas per total areas	13.03 ± 2.00	13.02 ± 1.52	12.90 ± 3.93	13.86 ± 1.90	0.917	0.754	0.754
PGP 9.5-positive cells per total cells	39.38 ± 4.64	36.14 ± 7.25	32.92 ± 6.50	37.31 ± 6.21	0.465	0.602	0.754
c-Kit–positive areas per total areas	0.95 ± 0.14	0.82 ± 0.21	0.40 ± 0.04	0.39 ± 0.06	**0.004**	**0.004**	**0.025**

The data are expressed as mean ± SD.

Bold style means the statistical significance.

nNOS, neuronal nitric oxide synthase; PGP, protein gene product

### Influence of aging on ICCs

**Fig 5:**
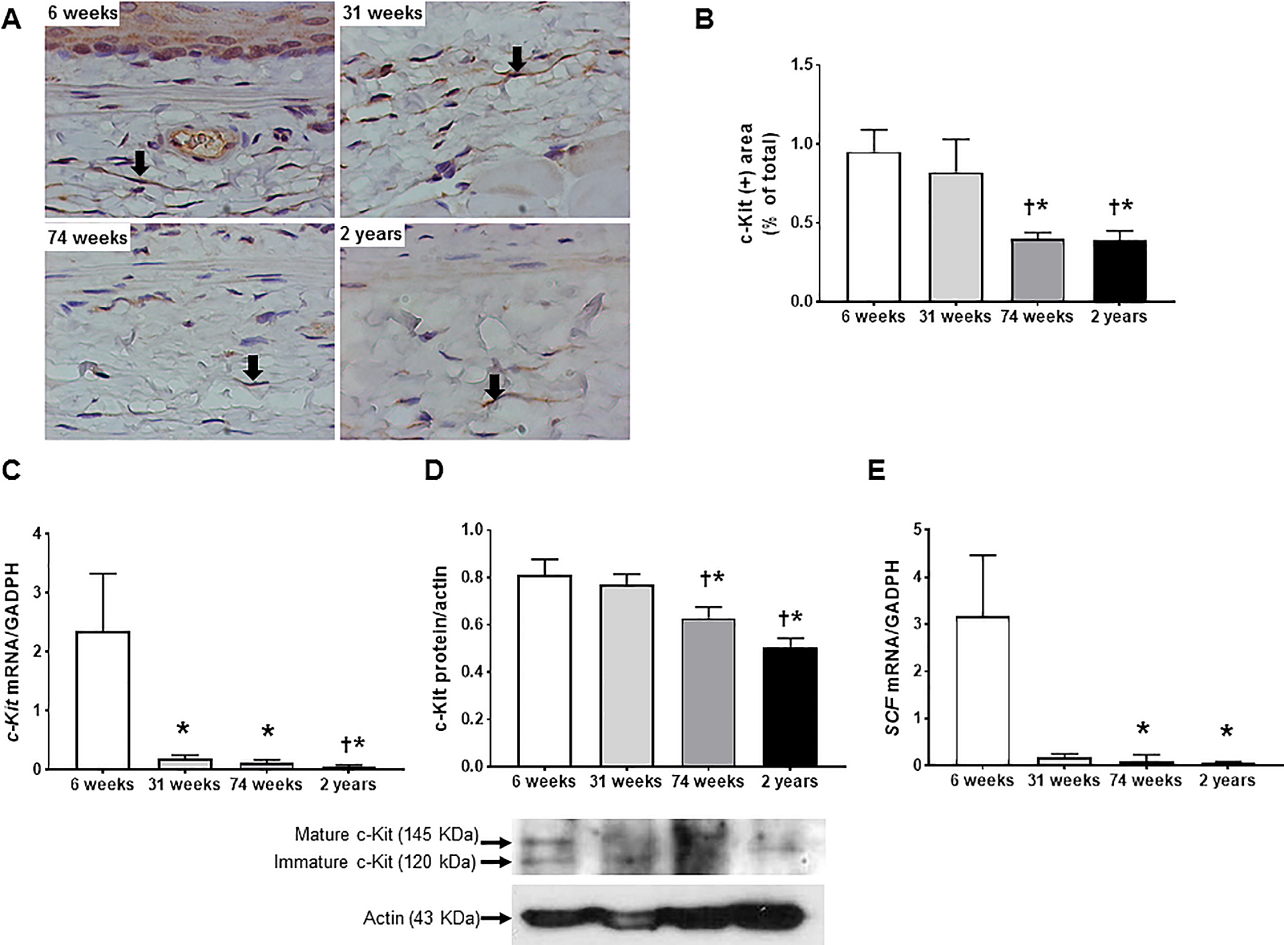
The change in c-Kit immunoreactivity and the expression of *c-Kit* mRNA, *stem cell factor* (*SCF*) mRNA and c-Kit protein of rat esophagus with aging. Representative images of c-Kit-immunoreactive cells in submucosal border of muscular layer (A), c-Kit immunohistochemistry (B) in 6-, 31-, 74-wk-, and 2-yr-old rats (each group, n = 6). Spindle shaped positively stained c-Kit-immunoreactive cells (arrow) distributed mainly in the submucosal border of muscular layer (x1000 magnification). c-Kit immunohistochemistry was expressed as percentage of c-Kit-immunoreactive areas per total area. Expression of *c-Kit* mRNA measured by real time-PCR (q-PCR) (C), the expression of c-Kit protein measured by western blot (D) and *SCF* mRNA measured by real time-PCR (E) in 6-, 31–74-wk-old rats (each, n = 7) and 2-yr-old rats (n = 10). Each bar represents the mean ± standard error. In western blot, the optical densities corresponding to the mature (145 kDa) and immature (120 kDa) forms were combined in the analysis process using densitometry.*P < 0.05 compared with 6 week of age; ^†^*P* < 0.05 compared with 31 week of age.

Fig 5A shows representative images of c-Kit-immunoreacitivity in the muscular layer of rat esophagus with four different ages. Spindle shaped positively stained c-Kit-immunoreactive cells dispersed mainly in the submucosal border of muscular layer, and there is no immunreactivity of c-Kit in the area of the ganglion cells of the myenteric plexus. Significantly lower proportion of c-Kit-immunoreactive area to total area was observed in the 74-wk- and 2-yr-old rats compared with the 6- and 31-wk-old rats (6-wk *vs* 74-wk, *P* = 0.004; 6-wk *vs* 2-yr, *P* = 0.004; 31-wk *vs* 74-wk, *P* = 0.037, 31-wk *vs* 2-yr, *P* = 0.025) (Table 2 and Fig 5B). *c-Kit* mRNA expression in the 74-wk- and 2-yr-old rats also decreased compared with that of the 6-wk-old rats (6-wk *vs* 74-wk, 2.35 ± 0.98 *vs* 0.12 ± 0.05, *P* = 0.013; 6-wk *vs* 2-yr, 2.35 ± 0.98 *vs* 0.06 ± 0.02, *P* = 0.001) (Fig 5C), the c-Kit protein expression in the 74-wk and 2-yr-old rats was lower compared with that of the 6-wk and 31-wk (6-wk *vs* 74-wk, 0.811 ± 0.066 *vs* 0.628 ± 0.047, *P* = 0.046; 6-wk *vs* 2-yr, 0.811 ± 0.066 *vs* 0.502 ± 0.042, *P* = 0.012; 31-wk *vs* 74-wk, 0.771 ± 0.044 *vs* 0.628 ± 0.047, *P* = 0.037; 31-wk *vs* 2-yr, 0.771 ± 0.044 *vs* 0.502 ± 0.042, *P* = 0.006) (Fig 5D). Similarly, *SCF* mRNA expressions in the 74-wk and 2-yr-old rats significantly decreased compared with that of the 6-wk-old rats (6-wk *vs* 74-wk, 3.18 ± 1.29 *vs* 0.09 ± 0.03, *P* = 0.025; 6-wk *vs* 2-yr, 3.18 ± 1.29 *vs* 0.07 ± 0.02, *P* = 0.006) (Fig 5E).

### Influence of aging on esophageal contractility

**Fig 6:**
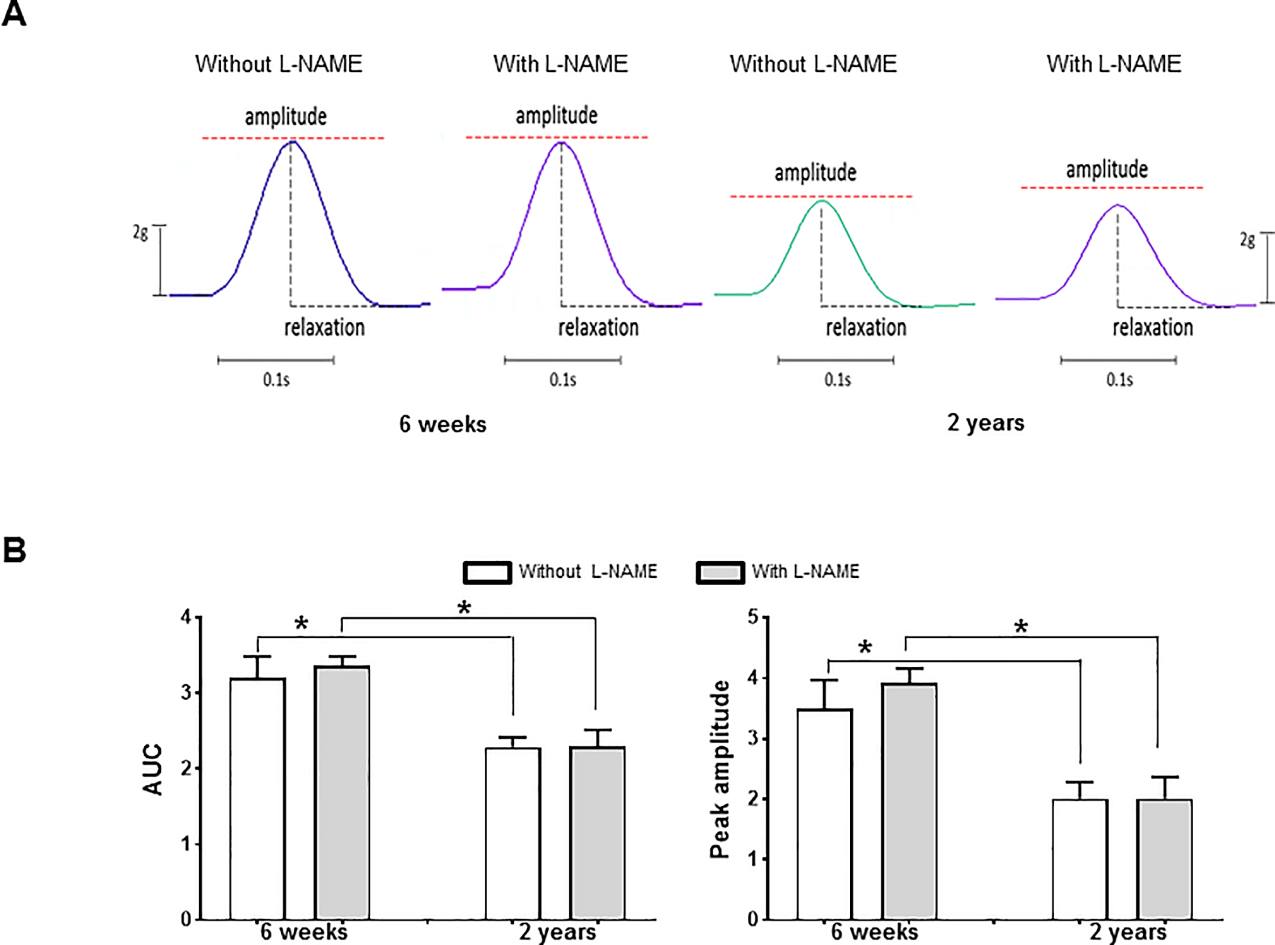
Single-pulse electrical field stimulation (EFS)- induced contractile response in the distal esophageal muscle. Representative traces (A) and area under curve (AUC) and peak amplitude (B) of single-pulse EFS-induced twitch-like contractions in the absence and presence of nitro-L-arginine methyl ester (L-NAME) in 6-wk- and 2-yr-old rats (each, n = 8). EFS was applied by using single pulses (voltage: 320 mV, duration: 0.3 ms). Each bar represents the mean ± standard error. *P < 0.05.

**Fig 7:**
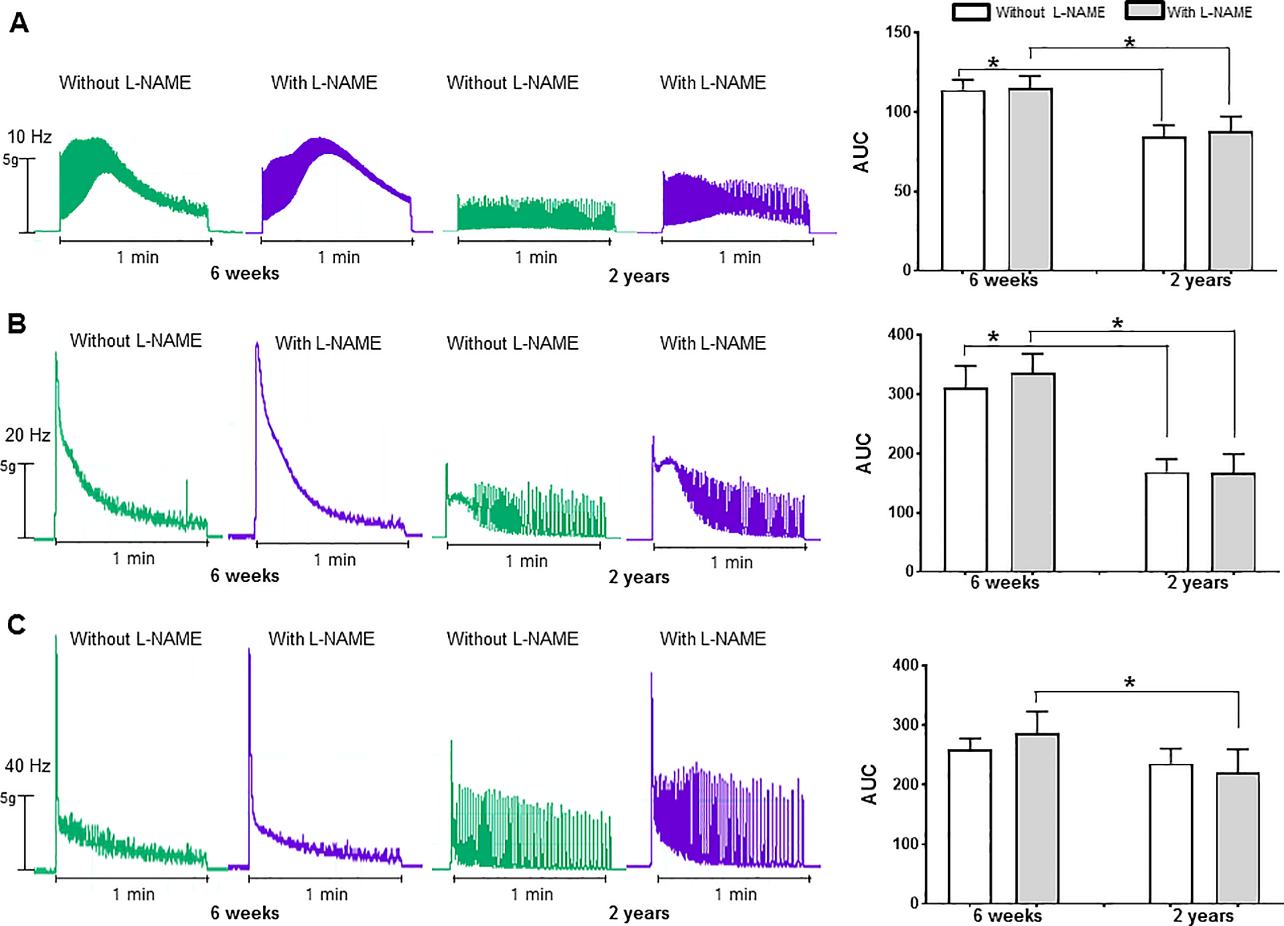
Multi-pulse electrical field stimulation (EFS)-induced contractile response in the distal esophageal muscle. Representative traces and area under curve (AUC) of contractile responses to multi-pulse EFS with 10 (A), 20 (B) and 40 Hz (C) in the absence and presence of nitro-L-arginine methyl ester (L-NAME) in 6-wk- and 2-yr-old rats (each, n = 8). Each bar represents the mean ± standard error. *P < 0.05.

When the spontaneous esophageal contractions after L-NAME treatment were compared with contractions before L-NAME which values were defined as 100%, L-NAME treatment did not significantly increase the contractions in the 6-wk-and 2-yr-old rat esophagus (6-wk-old rats, 100.51 ± 0.55%, *P* = 0.345; 2-yr-old-rats, 101.24 ± 0.63%, *P* = 0.068). The AUCs and peak amplitude of single-pulse and multi-pulse EFS-induced contractile responses of 2-yr-old rat esophagus were significantly decreased compared with the 6-wk-old rat esophagus in the absence of L-NAME (AUC, 2.29 ± 0.13 *vs* 3.20 ± 0.29, *P* = 0.014; amplitude, 2.01 ± 0.27 *vs* 3.50 ± 0.47, *P* = 0.021) (Figs 6 and 7). When we compared the AUC of contractions between the presence or absence of L-NAME, EFS-induced contractile responses tended to increase after administration of L-NAME in 6-wk-old rats, but there were no statistically significant differences (single-pulse, 3.20 ± 0.29 *vs* 3.37 ± 0.12, *P* = 0.674; multi-pulse with 10 Hz, 113.96 ± 6.36 *vs* 114.95 ± 7.81, *P* = 0.916; multi-pulse with 20 Hz, 311.70 ± 35.92 *vs* 335.30 ± 32.71, *P* = 0.345; multi-pulse with 40 Hz, 259.69 ± 18.55 *vs* 287.03 ± 221.16, *P* = 0.753) (Figs 6 and 7). In 2-yr-old rates, there were no significant differences of contractile responses to EFS between the presence and absence of L-NAME (single-pulse, 2.29 ± 0.13 *vs* 2.30 ± 0.22, *P* = 0.916; multi-pulse with 10 Hz, 84.63 ± 7.09 *vs* 88.04 ± 9.18, *P* = 0.916; multi-pulse with 20 Hz, 168.94 ± 21.81 *vs* 166.64 ± 32.57, *P* = 0.462; multi-pulse with 40 Hz, 235.88 ± 25.29 *vs* 221.16 ± 38.95, *P* = 0.345) (Figs 6 and 7).

## Discussions

In the present study, lamina propria thickness significantly increased with aging, which is similar to the finding of our previous research that showed an increase of lamina propria layer in the lower third of gastric F344 rats with aging [32]. It was accompanied by the increase in sulfated glycosaminoglycan and salt-soluble collagen which are major components of connective tissue [32]. The present study also showed a decrease in the endomysial area of LM during aging. The endomysial area of CM tended to decrease with aging, but there was no statistical significance. Endomysium is a layer of connective tissue that surrounds individual muscle cells tethering individual muscle fibers together, and functions as the sites for connections to cytoskeletal proteins of muscle cells. It is also source of extracellular materials including collagen and elastin, scaffolding for blood vessels. There might be an important influence on the functional and biologic properties of muscle cells because endomysium has the most intimate contact with the muscle cells [33]. The change in the thickness of lamina propria and endomysial area of muscular layer with aging might reflect the change of their components, influencing on esophageal function and motility as getting old. To the best of our knowledge, no research on endomysial area reported yet.

Our major finding is a loss of nNOS-immunoreactive cells and ICCs in old esophageal rat. PGP 9.5-immunoreactivity which is used to recognize the entire neuron did not significantly change with aging. And these cellular changes were also demonstrated in the molecular levels such as mRNA and protein in the present study. A loss of nitrergic neurons in the esophageal myenteric plexus has already been revealed in animal models of achalasia, as well as in patients with achalasia [34–36]. Achalasia is a primary motility disorder characterized by impaired LES relaxation and increased basal LES pressure with weak and simultaneous peristaltic waves in the esophageal body [37, 38]. It has also been shown that intramuscular ICCs were reduced and the ICC ultrastructure might be altered in humans with achalasia [34, 39]. In addition, nitric oxide (NO) has been shown to be the main inhibitory neurotransmitter responsible for esophageal function, especially relaxation of the LES [40]. NO generated by nNOS in inhibitory neurons results in the synthesis of cGMP by NO-sensitive guanylyl cyclase (NO-GC) in several target cells. NO-GC expression was detected in SMCs, ICCs, neurons and fibroblast-like cells in various parts of the GI tract [41]. The exact mechanism of nitrergic LES relaxation is still insufficiently elucidated. Recent experimental study using cell-specific knockout mouse lines for NO-GC suggested that the regulation of basal LES tone is based on a dual mechanism mediated by NO-GC in SMCs and ICCs whereas swallow-induced LES relaxation is mainly regulated by nitrergic mechanisms in ICCs [42]. Taken together, we suggest a selective loss of nNOS positive neuronal cells and ICCs with aging seems to cause impaired LES relaxation and peristalsis such as that observed in achalasia.

The results from our study are consistent to the age-related changes in stomach [20] and colon [19] of rats where a loss of ICCs and nNOS neurons in older rats was also observed. Subpopulations of ICCs are different between esophagus and other guts. The human esophagus has very few ICCs associated with the myenteric plexus and has abundant intramuscular ICCs dispersed throughout the circular and longitudinal muscle, while the ICCs of human stomach and colon are predominantly located both within the myenteric plexus and intramuscular structures [43]. We founded ICCs in the submucosal border of muscularis propria of rat esophagus, and our previous study showed that rat colon and stomach has ICCs predominantly in myenteric plexus and submucosal border [19, 20]. However, there is a limitation to use this animal model to explain the aging of human esophagus. The muscularis propria of the entire length of the esophagus in rodents such as rat contains only striated muscle [44]. On the contrary, the proximal half of the esophagus in human is composed of both striated and smooth muscles with predominance of striated muscle, whereas the distal part is composed of only smooth muscle [45]. Similar to our results, ICCs are scattered between striated muscle cells in the mouse esophagus [46]. Interestingly, ICCs are also found in the striated muscle of the human esophagus [47]. Despite of a difference of esophageal muscle type between humans and rats, rats have been used as animal models in basic research on esophageal function. Considering our data showing a reduction of ICCs in rat esophagus and a previous research reporting a reduction of ICCs in human stomach and colon with aging, ICCs of human esophagus might be decreased with aging. The change of ICCs influences on the esophageal function, the human study on this issue will be needed in the future. Nonetheless, our research has a strength that it is first to show histopathologic structures and expressions of ICCs and nNOS neurons in rat esophagus with different ages.

On the contrary to our study, several studies have presented the evidence that age-associated neuronal loss is specific to the cholinergic neurons in humans and rats, and nitrergic neurons are maintained [14–17]. Philipps *et al* reported that age-related cell loss in the myenteric plexus does not occur in nitrergic neurons; instead, it occurs exclusively in the cholinergic subpopulation of enteric neurons in Fischer 344 rats [15]. However, a decrease of nNOS expression with aging has been demonstrated in some studies on stomach [20] and colon [19] of F344 Fischer rats, and colon of Fisher (F344XBN) F1 rats [48]. It might be related to the damage of nitrergic neurons or a loss of expression of NOS in aged rat. Gamage *et al* showed the swollen and dystrophic processes of nNOS-immunoreactive neurons in aging mouse colon [17]. Our results suggest that the nitrergic neurons might be reduced in the number as shown in our study, or might undergo degenerative changes with aging even if they do not appear in detectable numbers at the ages studied. In addition, strain difference seems to exist in aging rat esophagus. We observed a loss of nNOS-immunoreactive cells in Fischer rats, while other group founded the reduced numbers of nitrergic neurons in Sprague Dawley rats, but maintained in Wistar rats [27].

In present study, esophageal peristalsis and LES function were not measured, our findings cannot directly be linked to impaired peristalsis and LES relaxation. Our functional study showed that EFS-induced contractile responses were significantly decreased in 2-yr-old rats compared with 6-wk-old rats. The pretreatment of L-NAME tended to increase the contractions in 6-yr-old rats and to induce no change in 2-yr-old-rats, but, disappointedly, the increments of contractile response to EFS in the presence of L-NAME were not statistically significant in both 6-wk- and 2-yr-old rats. Impaired esophageal contractility in the aged rats is due to impaired function of the esophageal striated cell themselves, or the disruption of the cells that regulate their activity such as neurons and ICCs. The esophageal striated muscle has known to receive dual innervation from both vagal motor fibers originating in brainstem and varicose intrinsic nerve fibers originating in myenteric plexus, which is called enteric co-innervation [49]. The exact contribution of the esophageal striated cells to esophageal motility and interaction with other cells such as ICCs are not sufficiently elucidated, yet. The changes of esophageal striated cells with aging were not investigated in present study. There has been only one early research reporting a decrease in the number and density of striated muscles in the proximal esophagus of elderly subjects [50]. The decrease of nNOS-postive cells and ICCs in the aged rat might influence esophageal contractions. Moreover, L-NAME is known as NO synthase inhibitor, the decrease of nNOS-positive cells in aged rat might cause less contractile change at the same dose of L-LAME in comparison to the young rats although there was no statistically significance.

The esophageal peristalsis is determined by a balance of intrinsic excitatory cholinergic and inhibitory nitrergic neurons. In current study, there were significant differences of EFS-induced contractile responses between 6-wk- and 2-yr-old rats, but L-NAME did not significantly affect the contractions in the 6-wk-and 2-yr-old rat esophagus. It might indicate that other inhibitory neurotransmitters (neuropeptide Y, vasoactive intestinal peptide, etc) or excitatory cholinergic neurons play a role in contractile differences between young and aged rat esophagus. As we focused on the change of nitrergic neurons with age, the research on the change of excitatory neurons or other inhibitory neurons with age is needed in the future study. It is a limitation of the present study.

In conclusion, our data clearly showed that aging induces an increase of lamina propria thickness, a decrease of LM endomysial area, and reduced expressions of nNOS, c-Kit and SCF in the rat esophagus. These cellular changes linked to decreased esophageal contractility in aged rats. The future study using esophageal manometry is needed to age-related cellular changes to be correlated to impaired peristalsis and LES relaxation.
